# Assessment of Fringe Pattern Decomposition with a Cross-Correlation Index for Phase Retrieval in Fringe Projection 3D Measurements

**DOI:** 10.3390/s18103578

**Published:** 2018-10-22

**Authors:** Xinjun Zhu, Limei Song, Hongyi Wang, Qinghua Guo

**Affiliations:** 1 Key Laboratory of Advanced Electrical Engineering and Energy Technology, Tianjin Polytechnic University, Tianjin 300387, China; wanghongyi@tjpu.edu.cn (H.W.); qguo@uow.edu.au (Q.G.); 2 School of Electrical, Computer and Tele communications Engineering, University of Wollongong, Wollongong, NSW 2500, Australia

**Keywords:** fringe pattern decomposition, cross-correlation, phase retrieval, Fourier transform, Shearlet transform, parameter selection

## Abstract

Phase retrieval from single frame projection fringe patterns, a fundamental and challenging problem in fringe projection measurement, attracts wide attention and various new methods have emerged to address this challenge. Many phase retrieval methods are based on the decomposition of fringe patterns into a background part and a fringe part, and then the phase is obtained from the decomposed fringe part. However, the decomposition results are subject to the selection of model parameters, which is usually performed manually by trial and error due to the lack of decomposition assessment rules under a no ground truth data situation. In this paper, we propose a cross-correlation index to assess the decomposition and phase retrieval results without the need of ground truth data. The feasibility of the proposed metric is verified by simulated and real fringe patterns with the well-known Fourier transform method and recently proposed Shearlet transform method. This work contributes to the automatic phase retrieval and three-dimensional (3D) measurement with less human intervention, and can be potentially employed in other fields such as phase retrieval in digital holography.

## 1. Introduction

Fringe projection optical three-dimensional (3D) shape measurement methods are becoming more and more popular in recent years due to their ability to provide high-resolution, high-speed, whole-field 3D reconstruction of objects in a non-contact manner. They have been extensively investigated and widely used in numerous fields such as industrial and scientific, biomedical, kinematics, biometric identification, and cultural heritage and preservation applications [1,2,3,4,5]. Phase retrieval is a key step in fringe projection measurement, and is of fundamental importance to the successful application of the method [1,6]. Phase retrieval can be achieved from multiple frame fringe patterns with well-known phase shift algorithms or from single frame fringe patterns with the well-known Fourier transform method [7,8]. In the measurement of objects in fast motion or in a temporally unstable environment, it is difficult or costly (e.g., using a high speed camera) to take several fringe projection patterns in an extremely short period of time. Therefore, phase retrieval based on a single frame fringe projection pattern is highly desirable in these cases.

By now, phase retrieval from a single frame fringe projection pattern has been extensively studied. Interested readers may refer to [1] for a comprehensive review of phase retrieval in fringe projection profilometry. The Fourier transform method and window Fourier transform (WFT) method are two widely used and well-known single frame phase retrieval techniques [8,9]. In addition, the wavelet transform method and the more recently developed empirical mode decomposition (EMD), variational image decomposition (VID) and Shearlet transform-based methods [10,11,12,13] have been proposed for phase retrieval from a single projection fringe pattern. Although the principles of these methods are different, many of them rely on background elimination, which can be formulated as a fringe pattern decomposition problem.

Parameter selection in single frame projection fringe pattern phase retrieval is important but has received less attention. For instance, the Fourier transform-based fringe decomposition and corresponding phase retrieval performance are related to the filtering window size [8]. Shearlet transform decomposition and corresponding phase retrieval results are related to the decomposition layer [13]. An inappropriate parameter value may degrade the decomposition results. Therefore, it is important to choose an appropriate value of model parameter to produce desirable results. Usually, the optimal parameter selection is conducted manually by trial and error with lots of experiments due to the lack of decomposition assessment rules.

In this paper, we propose a cross-correlation criteria to assess the fringes and background decomposition for automatically selecting the optimal parameter of Fourier transform and Shearlet transform-based fringe pattern decomposition methods. The proposed cross-correlation index is calculated by using the decomposed background part and fringe part, and thus it does not require the ground truth data. The contribution of the paper is twofold: first, the cross-correlation index to assess the decomposition results of fringe pattern is proposed and verified to be simple but feasible. Second, the proposed cross-correlation metric is suitable for the Fourier transform and Shearlet transform parameters selection and maybe extended to other phase retrieval methods such as WFT and EMD. The organization of this paper is as follows: in Section 2, a brief introduction of Fourier transform and Shearlet transform method with corresponding parameter descriptions is presented. After that, the cross-correlation metric of the decomposed background and fringe is proposed. In Section 3, the proposed cross-correlation metric is verified by simulated and experimental data and results discussion are given. Section 4 concludes the paper.

## 2. Cross-Correlation of Background Part and Fringe Part for Fourier Transform and Shearlet Transform Methods

### 2.1. Fourier Transform and Shearlet Transform Methods Based Fringe Pattern Decomposition

#### 2.1.1. Fourier Transform Method for Fringe Projection

In fringe projection, a fringe pattern captured by a CCD can be expressed as:

(1) $$I\left(x,y\right)=a\left(x,y\right)+b\left(x,y\right)\cos\left(\phi \left(x,y\right)+2\pi f_{0}x\right),$$

where a(x,y) is the background, b(x,y) and ϕ(x,y) are the modulation intensity and the optical phase, and $f_{0}$ is carrier frequency. Equation (1) can be rewritten in complex form

(2) $$I\left(x,y\right)=a\left(x,y\right)+c\left(x,y\right)\exp(j2\pi f_{0}x)+c^{*}\left(x,y\right)\exp(-j2\pi f_{0}x),$$

where $c\left(x,y\right)=\frac{b(x,y)}{2}\exp\left(j\left(\phi (x,y)\right)\right)$, and $c^{*}\left(x,y\right)$ denotes the complex conjugate.

The Fourier transform of fringe pattern I(x,y) with frequency $f_{0}$ is comprised by spectrum components separated from each other:

(3) $$\hat{I}\left(v_{x},v_{y}\right)=A\left(v_{x},v_{y}\right)+C\left(v_{x}-f_{0},v_{y}\right)+C^{*}\left(\left(v_{x}+f_{0}\right),v_{y}\right),$$

where $\hat{I}\left(v_{x},v_{y}\right)$ is the Fourier transform of fringe pattern I(x,y) with frequency coordinate $\left(v_{x},v_{y}\right)$, $A\left(v_{x},v_{y}\right)$ is the Fourier transform of a(x,y), $C\left(v_{x},v_{y}\right)$ is the Fourier transform of c(x,y) and $C^{*}\left(v_{x},v_{y}\right)$ is Fourier transform of $c^{*}\left(x,y\right)$. $A\left(v_{x},v_{y}\right)$ denotes the Fourier transform of background part, $C\left(v_{x}-f_{0},v_{y}\right)+C^{*}\left(\left(v_{x}+f_{0}\right),v_{y}\right)$ denotes the Fourier transform of fringe part. This allows selectively filtering with a window to obtain the background spectrum component $A\left(v_{x},v_{y}\right)$ or the fringe spectrum component $C\left(v_{x}-f_{0},v_{y}\right)+C^{*}\left(\left(v_{x}+f_{0}\right),v_{y}\right)$. Then by applying the inverse Fourier transform, one can obtain the background part and fringe part. The fringe part is:

(4) $$Fringe=F^{-1}\left(C\left(v_{x}-f_{0},v_{y}\right)+C^{*}\left(\left(v_{x}+f_{0}\right),v_{y}\right)\right)$$

where $F^{-1}$ denotes inverse Fourier transform. The decomposition results are depended on the Fourier transform parameter (filtering window size), which should be carefully selected.

#### 2.1.2. Shearlet Transform Method for Fringe Projection

In the Shearlet transform method, firstly, a forward Shearlet transform is performed on *I*(*x*,*y*) to obtain the transform coefficients:

(5) $$SH_{I}=SH\left\{I\left(x,y\right)\right\},$$

where SH denotes forward Shearlet transform operator on image *I*(*x*,*y*); secondly, the transform coefficients undergo a hard thresholding before an inverse Shearlet transform is carried out [13]. Thirdly, an inverse Shearlet transform will operate on the remaining resultant Shearlet coefficients to obtain the fringe part:

(6) $$Fringe=SH^{-1}\left\{SH_{I}\left\{i\right\}\right\},i=1,2,3,4;$$

where *i* is the decomposition scale, and *SH*^−1^ is inverse Shearlet transform. In this study, we investigate the cross-correlation of the background part and fringe part. Therefore, we set the thresholding with threshold value of zero and set the Shearlet coefficients of the first scale of $SH_{I}$ to be zero. By these, the background of a projection fringe pattern can be removed, and the fringe part can be obtained. The Shearlet transform parameter (decomposition layer *i*) needs to be carefully selected.

With the obtained fringe v(x,y) in Equation (4) or Equation (6), the phase distribution with carrier is calculated as follows:

(7) ϕ(x,y)+φc(x,y)=arctan(Im{H(v(x,y))}Re{H(v(x,y))}).

where *H* denotes the Hilbert transform, Re{ } and Im{ } respectively denote the real and imaginary parts, and $\varphi_{c}\left(x,y\right)$ is the carrier which should be removed to produce a pure phase.

### 2.2. The Proposed Cross-Correlation Index for Decomposition Assessment

As mentioned above, the decomposition result depends on the parameter selection and the result assessment requires an assessment index. It can be supposed that fringe pattern *f* = *u* + *v* is decomposed into background *u* and fringe *v*. Background varies slowly, while fringe exhibits texture feature. Here we assume that the background part and fringe part are highly uncorrelated or lowly correlated and that they have small cross-correlation distance metric. Given the background part *u* and fringe part *v*, the cross-correlation distance metric (CrossUV) is:

(8) $$CrossUV=\frac{\sum_{m,n}^{M,N}\left(u-\bar{u}\right)\left(v-\bar{v}\right)}{\sqrt{\sum_{m,n}^{M,N}{\left(u-\bar{u}\right)}^{2}\sum_{m,n}^{M,N}{\left(v-\bar{v}\right)}^{2}}}$$

where $\bar{u}$ and $\bar{v}$ are the mean values of *u* and *v* respectively, *M* and *N* are the sizes of *u*.

The error of the finally unwrapped phase is:

(9) $$SE=\frac{\sum_{i=1}^{M}\sum_{j=1}^{N}{\left[\psi \left(i,j\right)-\psi_{0}\left(i,j\right)\right]}^{2}}{\sum_{i=1}^{M}\sum_{j=1}^{N}{\left[\psi_{0}\left(i,j\right)\right]}^{2}}$$

where ψ(i,j) and $\psi_{0}\left(i,j\right)$ are the retrieved and theoretical phase values, respectively.

The Structural Similarity (*SSIM*) metric is calculated on decomposed fringe *x* and referenced fringe *y*, which is:

(10) $$SSIMV(x,y)=\frac{\left(2\mu_{x}\mu_{y}+c_{1})(2\sigma_{xy}+c_{2}\right)}{\left(\mu_{x}^{2}+\mu_{y}^{2}+c_{1})(\sigma_{x}^{2}+\sigma_{y}^{2}+c_{2}\right)}$$

where $\mu_{x}$, $\mu_{y}$, $\sigma_{x}$, $\sigma_{y}$ and $\sigma_{xy}$ are the local means, standard deviations, and cross-covariance for images *x* and *y*, and $c_{1}$ and $c_{2}$ are regularization constants [14].

## 3. Experimental Results and Discussions

**Figure 1:**
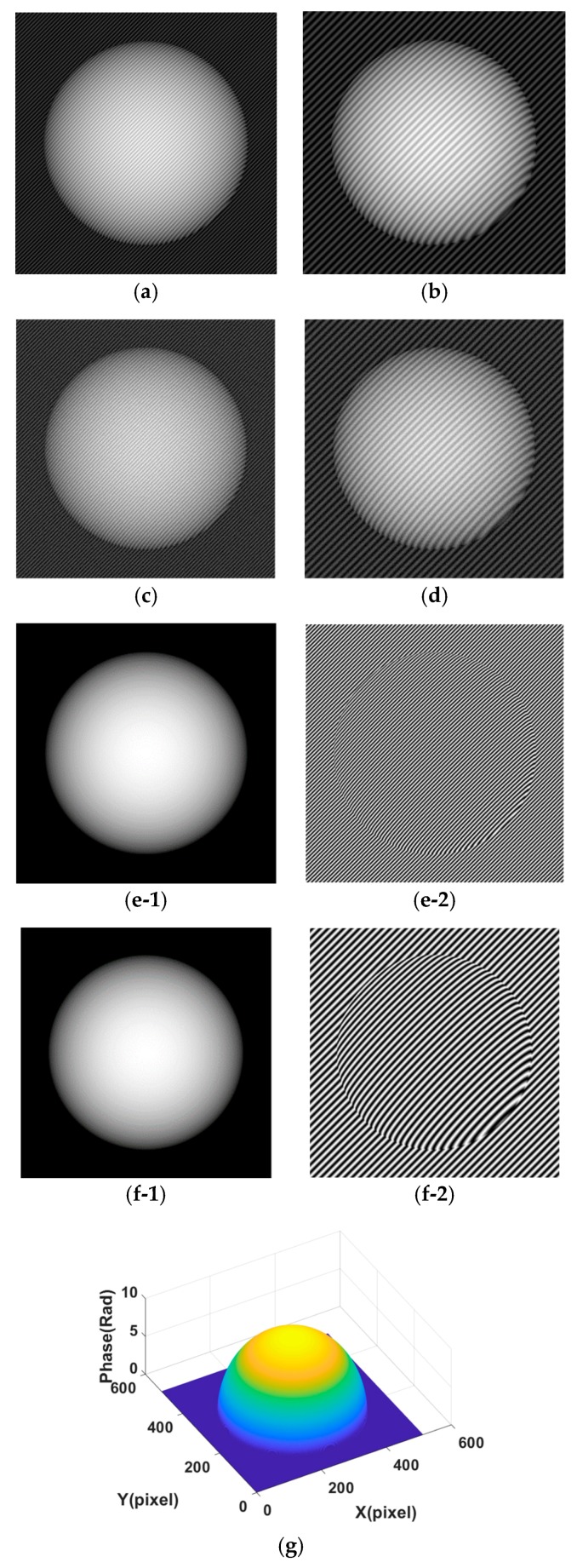
Simulated projection fringe patterns of sphere shape. (**a**) Fringe pattern with frequency 1/8 without noise added; (**b**) Fringe pattern with frequency 1/16 without noise added; (**c**) Fringe pattern with frequency 1/8 with Gaussian noise added; (**d**) Fringe pattern with frequency 1/16 with Gaussian noise added; (**e-1**) The ground truth background part of Figure 1a,c; (**e-2**) The ground truth fringe part of Figure 1a,c; (**f-1**) The ground truth background part of Figure 1b,d; (**f-2**) The ground truth fringe part of Figure 1b,d; (**g**) The ground truth phase.

**Figure 2:**
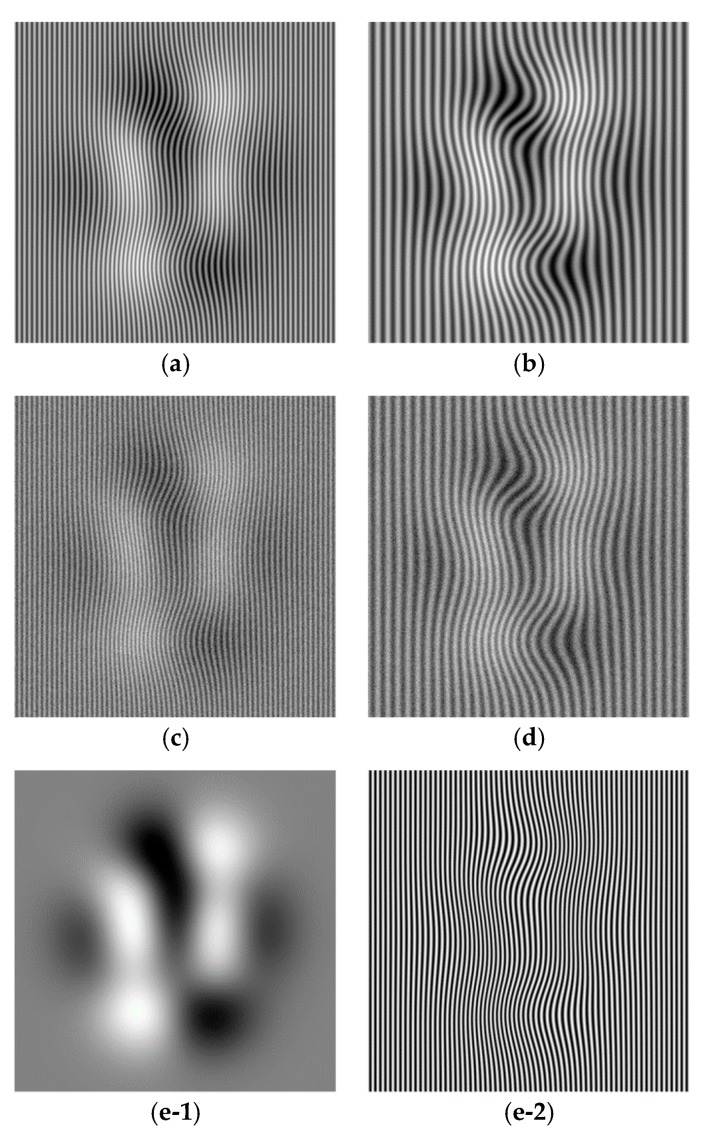

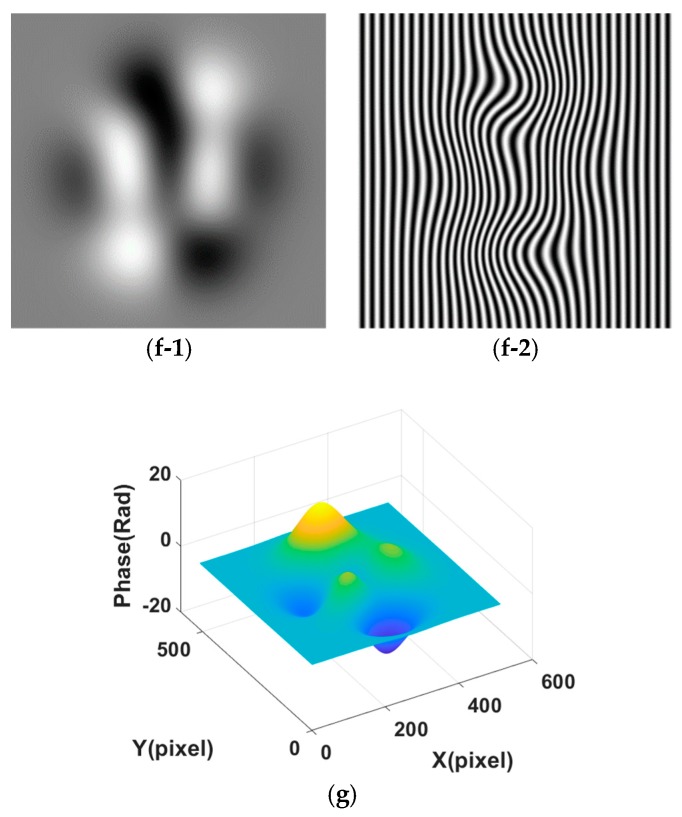
Simulated projection fringe patterns of peaks shape. (**a**) Fringe pattern with frequency 1/8 without noise added; (**b**) Fringe pattern with frequency 1/16 without noise added; (**c**) Fringe pattern with frequency 1/8 with Gaussian noise added; (**d**) Fringe pattern with frequency 1/16 with Gaussian noise added; (**e-1**) The ground truth background part of Figure 2a,c; (**e-2**) The ground truth fringe part of Figure 2a,c; (**f-1**) The ground truth background part of Figure 2b,d; (**f-2**) The ground truth fringe part of Figure 2b,d; (**g**) The ground truth phase.

**Figure 3:**
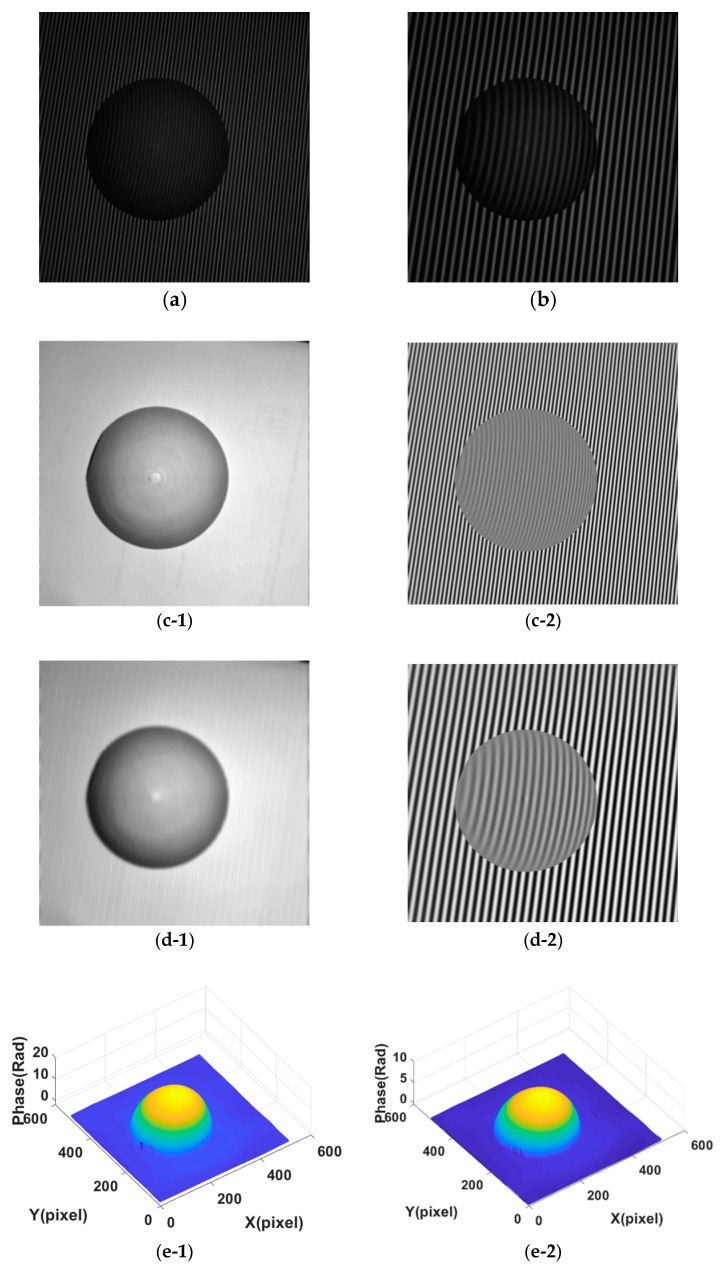
Experimental projection fringe patterns. (**a**) Fringe pattern with larger frequency; (**b**) Fringe pattern with small frequency. (**c-1**) The ground truth background part of Figure 3a; (**c-2**) The ground truth fringe part of Figure 3a; (**d-1**) The ground truth background part of Figure 3b; (**d-2**) The ground truth fringe part of Figure 3b; (**e-1**) The ground truth phase for Figure 3a; (**e-2**) The ground truth phase for Figure 3b.

Next, we use the cross-correlation metric to test the relation of background and fringe decomposed from simulated and real fringe projection patterns. In this study, the well-known Fourier transform method and recently proposed Shearlet transform method are employed to decompose the fringe projection pattern. The adopted fringe patterns are respectively shown in Figure 1, Figure 2 and Figure 3. Figure 1a,b are simulated fringe pattern with different carry frequencies. The simulated projection fringe patterns of a sphere shape with abrupt changes (Figure 1) with the sizes of 512 × 512 pixels are generated by:

(11) $$I\left(x,y\right)=a\left(x,y\right)+b\left(x,y\right)\cos\left(\phi \left(x,y\right)+2\pi f_{0}\left(x+y\right)\right)$$

with phase:

(12) ϕ(x,y)=Re{10(1−(x−256)2+(y−256)22002)},

where Re{ } denotes the real part. The spatial frequency of the fringe pattern is set to *f*_0_ = 1/8 for Figure 1a and *f*_0_ = 1/16 for Figure 1b, and the background illumination *a*(*x*,*y*) is 0.5×ϕ(x,y) which makes the background outside of object region different to the background inside of test object, the modulation intensity b(x,y) is 1. Figure 1c,d show the noisy fringe patterns corresponding to Figure 1a,b with Gaussian random noise with variance of 0.2 added [12]. In addition, Figure 1(e-1) shows the ground truth background part of Figure 1a,c; Figure 1(e-2) shows the ground truth fringe part of Figure 1a,c; Figure 1(f-1) shows the ground truth background part of Figure 1b,d; Figure 1(f-2) shows the ground truth fringe part of Figure 1b,d; Figure 1g shows the ground truth phase.

Similarly, Figure 2a,b are simulated fringe pattern with smooth changes with the sizes of 512 × 512 pixels. They are generated by:

(13) $$I\left(x,y\right)=a\left(x,y\right)+b\left(x,y\right)\cos\left(2\times peaks\left(x,y\right)+2\pi f_{0}x\right)$$

where peaks(x,y) is the peaks function in Matlab (Mathworks, Natick, MA, USA), a(x,y)=5×δ(peaks(x,y))/δx, b(x,y)=0.5. The carrier frequency $f_{0}$ for Figure 2a,b are respectively 1/8 and 1/16. Figure 2c,d show the noisy fringe patterns corresponding to Figure 2a,b with Gaussian random noise with variance of 0.2 added [11,12,13]. In addition, Figure 2(e-1) shows the ground truth background part of Figure 2a,c; Figure 2(e-2) shows the ground truth fringe part of Figure 2a,c; Figure 2(f-1) shows the ground truth background part of Figure 2b,d; Figure 2(f-2) shows the ground truth fringe part of Figure 2b,d; Figure 2g shows the ground truth phase.

Figure 3 shows experimental fringe projection patterns with image sizes of 512 × 512 pixels, which depicts a model of plastic sphere. Figure 3a is with a large frequency while Figure 3b is with a small frequency. Figure 3(c-1) shows the ground truth background part of Figure 3a; Figure 3(c-2) shows the ground truth fringe part of Figure 3a; Figure 3(d-1) shows the ground truth background part of Figure 3b; Figure 3(d-2) shows the ground truth fringe part of Figure 3b; Figure 3(e-1) shows the ground truth phase for Figure 3a; Figure 3(e-2) shows the ground truth phase for Figure 3b. For the experimental fringe projection patterns, we use one projector (DLP LightCrafter 3000, TI, Dallas, TX, USA) with resolution of 608 × 684 to project sinusoidal fringe pattern and gray scale CCD camera (SXG10, Baumer, Frauenfeld, Switzerland) with recording resolution of 1024 × 1024 pixels.

Fringe patterns are analyzed as follows: The fringe patterns are decomposed by the Fourier transform and Shearlet transform methods respectively to give the decomposed background (part) *u* and fringe (part) *v*, i.e., *I* = *u* + *v*. In the decomposition, in order to test the effect of parameter on decomposition results, the parameter for the Fourier transform takes a range of values 2:1:20 (from 2 to 20 with increment 1, denoted as P1) for Figure 1a, 4:2:30 (denoted as P2) for Figure 1b, 2:1:20 for Figure 1c, 4:2:30 for Figure 1d, 4:1:20 for Figure 2a, 5:3:50 for Figure 2b, 4:1:20 for Figure 2c, 6:3:50 for Figure 2d, 4:1:20 for Figure 3a, 6:2:40 for Figure 3b. The parameter values for Shearlet transform are set as decomposition layer 3 and 4. The cross-correlation of the background part and fringe part is calculated by Equation (8). The error and SSIM of fringe part are calculated by Equations (9) and (10) respectively.

**Figure 4:**
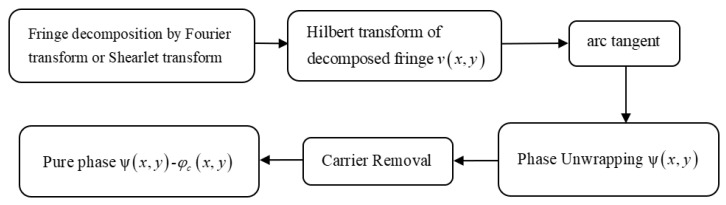
The diagram of phase retrieval by Fourier transform and Shearlet transform.

Figure 4 shows the diagram of phase retrieval of Fourier transform and Shearlet transform. With the derived fringe, the wrapped phase is obtained by Hilbert transform and arc tangentatan operator on the decomposed fringe part. Further, the unwrapped phases were obtained by quality guided phase unwrapping algorithm [15]. To obtain the pure unwrapped phases without the carrier term, the carrier was removed from the unwrapped phases by Zernike fitting method [16]. To sum up, the decomposed fringes, and unwrapped phase are obtained, from which the assessment indexes of error and SSIM are calculated to give overall assessment of decomposition results. To use the true data in the assessment of experimental fringe pattern, the fringes part and unwrapped phase by four steps phase shift method are considered as the true data [12].

**Figure 5:**
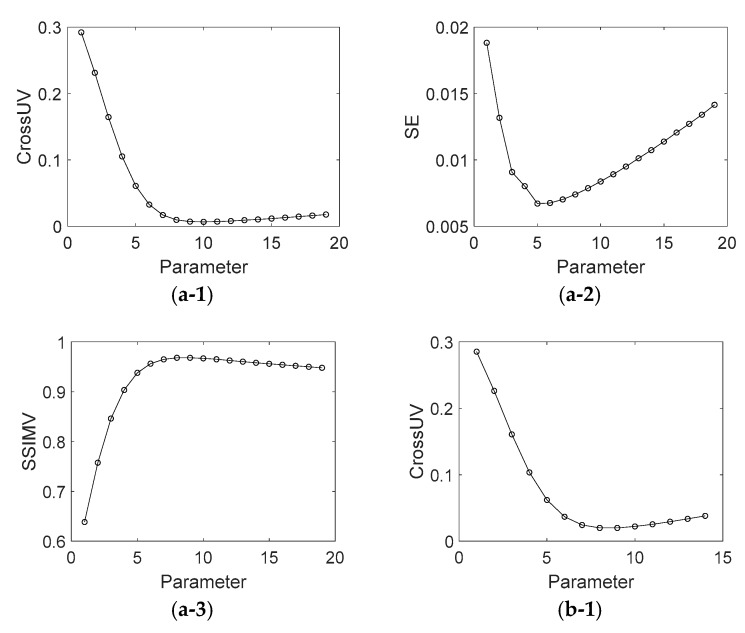

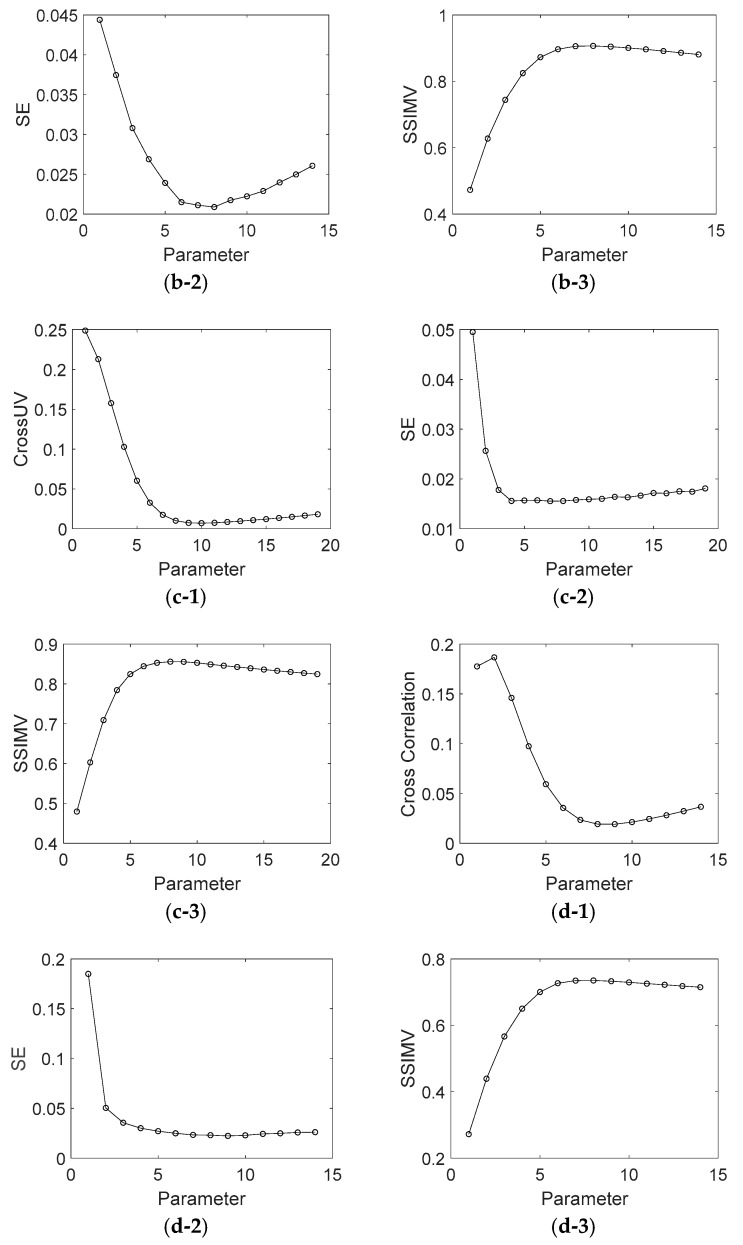
CrossUV, SE and SSIMV for Figure 1 by Fourier transform method with different parameter values. (**a-1**) CrossUV for Figure 1a; (**a-2**) SE for Figure 1a; (**a-3**) SSIMV for Figure 1a; (**b-1**) CrossUV for Figure 1b; (**b-2**) SE for Figure 1b; (**b-3**) SSIMV for Figure 1b. (**c-1**) CrossUV for Figure 1c; (**c-2**) SE for Figure 1c; (**c-3**) SSIMV for Figure 1c; (**d-1**) CrossUV for Figure 1d; (**d-2**) SE for Figure 1d; (**d-3**) SSIMV for Figure 1d.

**Figure 6:**
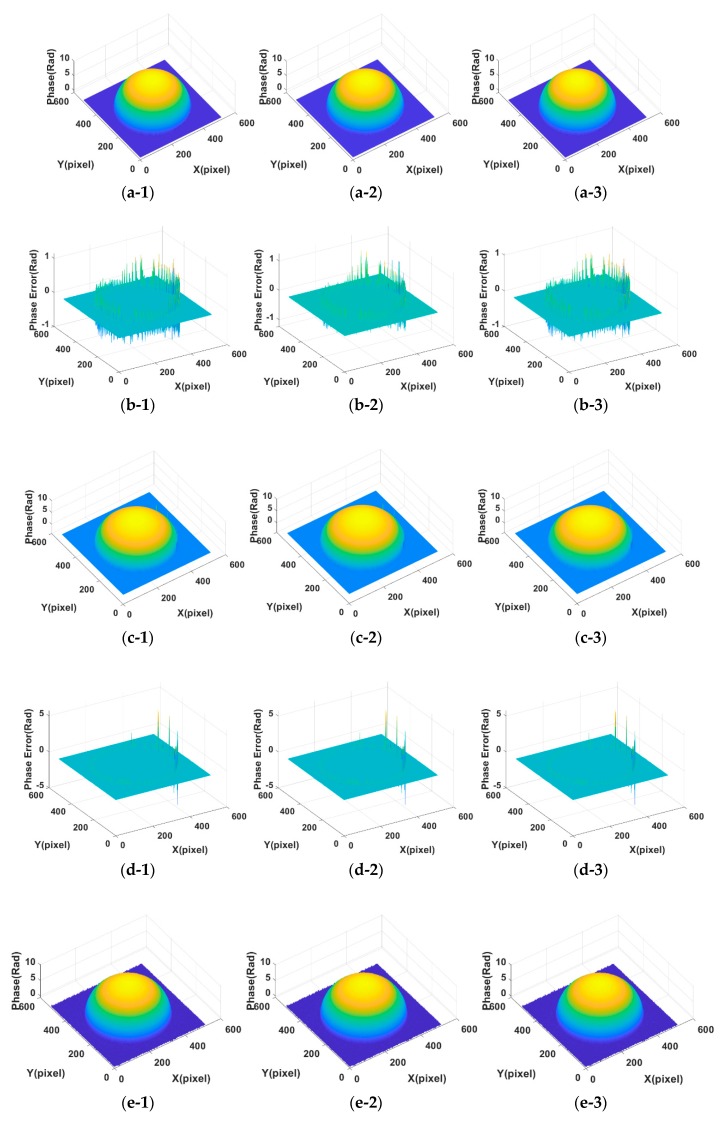

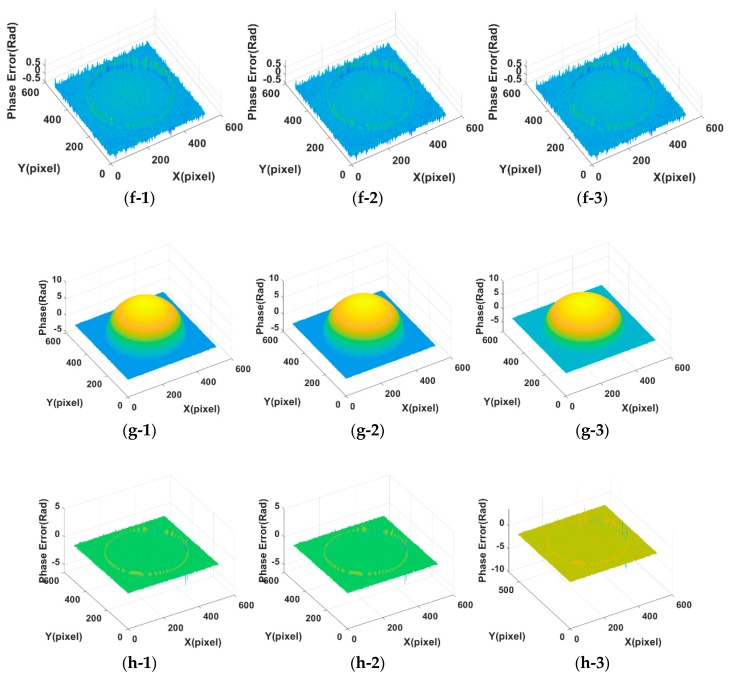
The retrieved phase and phase error for Figure 1 by Fourier transform method under optimal CrossUV, SE and SSIMV. (**a-1**) Phase under optimal CrossUV for Figure 1a; (**a-2**) Phase under optimal SE for Figure 1a; (**a-3**) Phase under optimal SSIMV for Figure 1a; (**b-1**) Phase error under optimal CrossUV for Figure 1a; (**b-2**) Phase error under optimal SE for Figure 1a; (**b-3**) Phase error under optimal SSIMV for Figure 1a; (**c-1**) Phase under optimal CrossUV for Figure 1b; (**c-2**) Phase under optimal SE for Figure 1b; (**c-3**) Phase under optimal SSIMV for Figure 1b; (**d-1**) Phase error under optimal CrossUV for Figure 1b; (**d-2**) Phase error under optimal SE for Figure 1b; (**d-3**) Phase error under optimal SSIMV for Figure 1b; (**e-1**) Phase under optimal CrossUV for Figure 1c; (**e-2**) Phase under optimal SE for Figure 1c; (**e-3**) Phase under optimal SSIMV for Figure 1c; (**f-1**) Phase error under optimal CrossUV for Figure 1c; (**f-2**) Phase error under optimal SE for Figure 1c; (**f-3**) Phase error under optimal SSIMV for Figure 1c; (**g-1**) Phase under optimal CrossUV for Figure 1d; (**g-2**) Phase under optimal SE for Figure 1d; (**g-3**) Phase under optimal SSIMV for Figure 1d; (**h-1**) Phase error under optimal CrossUV for Figure 1d; (**h-2**) Phase error under optimal SE for Figure 1d; (**h-3**) Phase error under optimal SSIMV for Figure 1d.

**Figure A1:**
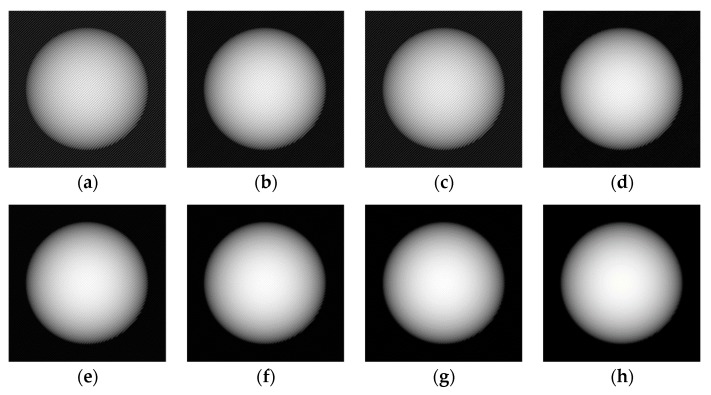

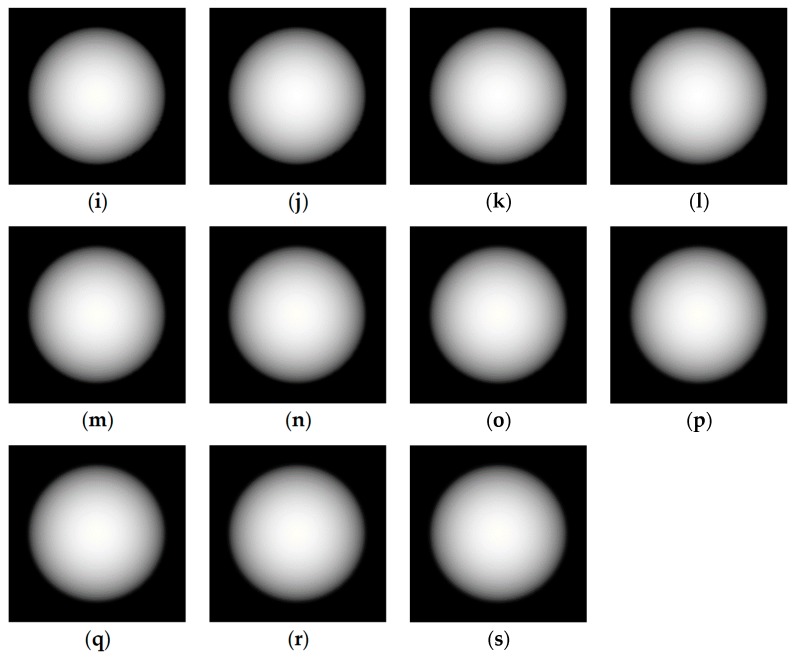
The decomposed background of Figure 1a by Fourier transform method with different parameter values. (**a**–**s**) are the decomposed background by Fourier transform method with 1th to 19th of filtering window sizes of [2:1:20], respectively.

**Figure A2:**
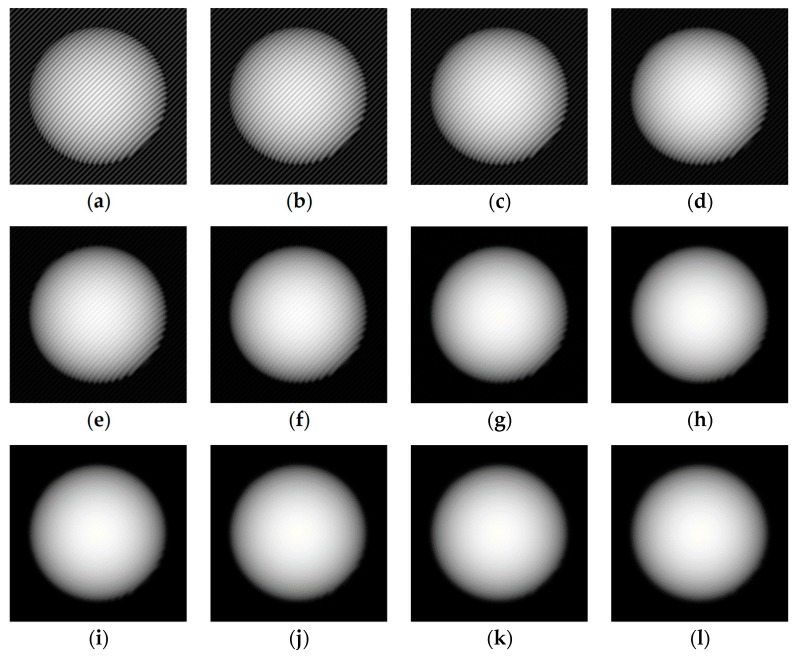

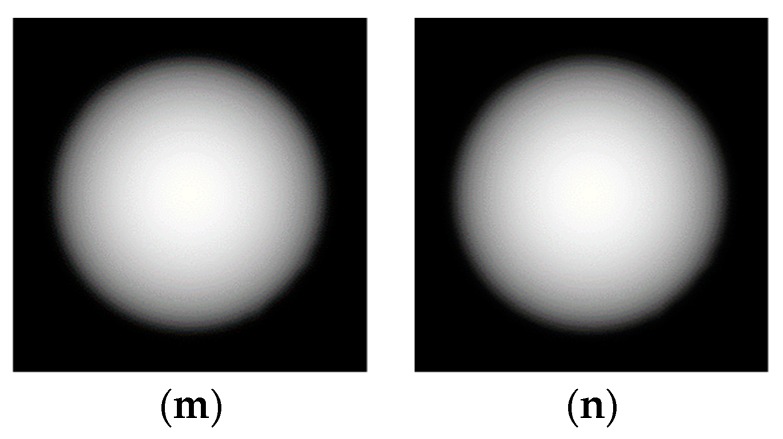
The decomposed background of Figure 1b by Fourier transform method with different parameter values. (**a**–**n**) are the decomposed background by Fourier transform method with 1th to 14th of filtering window sizes of [4:2:30], respectively.

In order to show the effect of parameter values on the decomposition results of Figure 1 in terms of visual quality, the decomposition background parts from Figure 1a,b under different parameter values are shown in Figure A1 and Figure A2 (See Appendix A). Figure 5 shows the assessment index of CrossUV, SE and SSIMV for simulated fringe patterns (Figure 1) by Fourier transform method under a set of model parameter values. Specifically, Figure 5(a-1)–(a-3) respectively show the CrossUV, SE and SSIMV for Figure 1a; Figure 5(b-1)–(b-3) respectively show the CrossUV, SE and SSIMV for Figure 1b; Figure 5(c-1)–(c-3) respectively show the CrossUV, SE and SSIMV for Figure 1c; Figure 5(d-1)–(d-3) respectively show the CrossUV, SE and SSIMV for Figure 1d. Figure 6 shows the retrieved phase and phase error for Figure 1 by the Fourier transform method under optimal CrossUV, SE and SSIMV.

**Figure 7:**
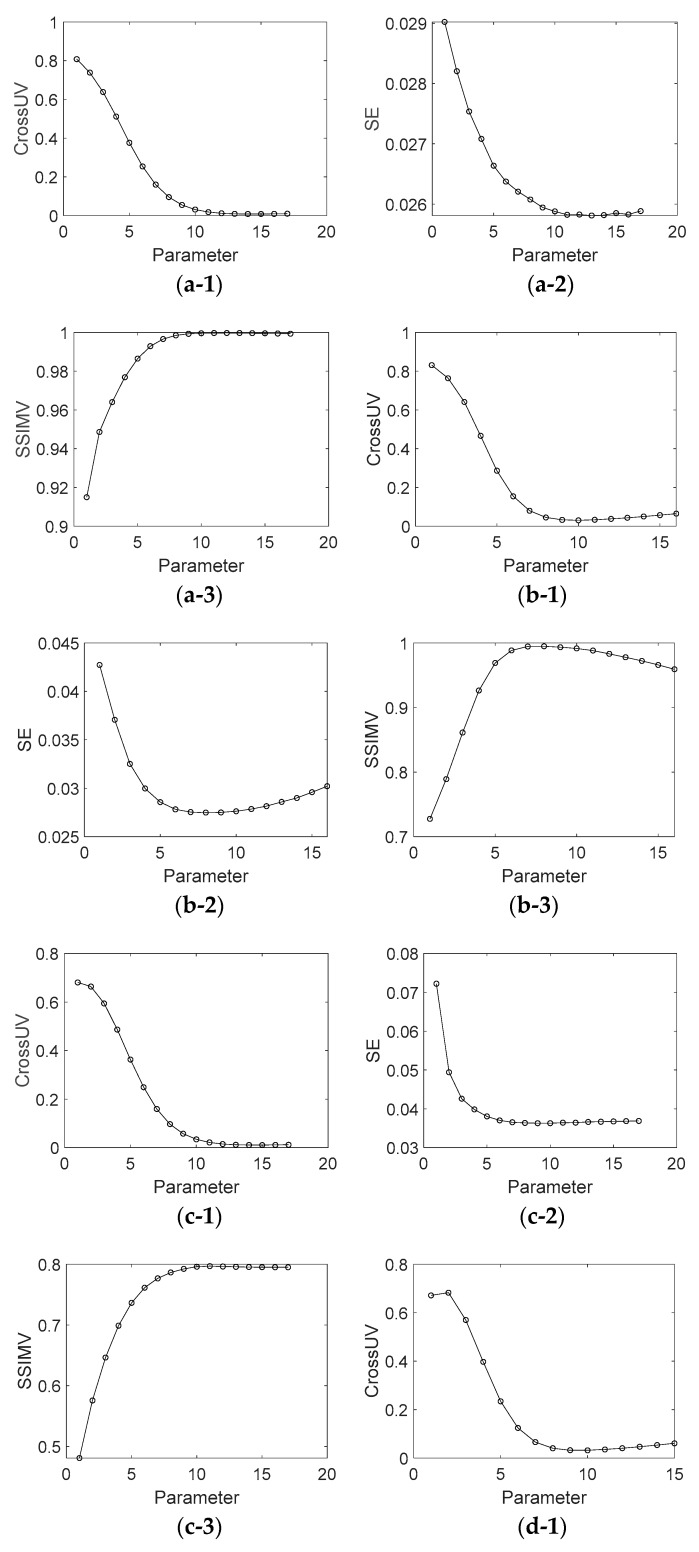

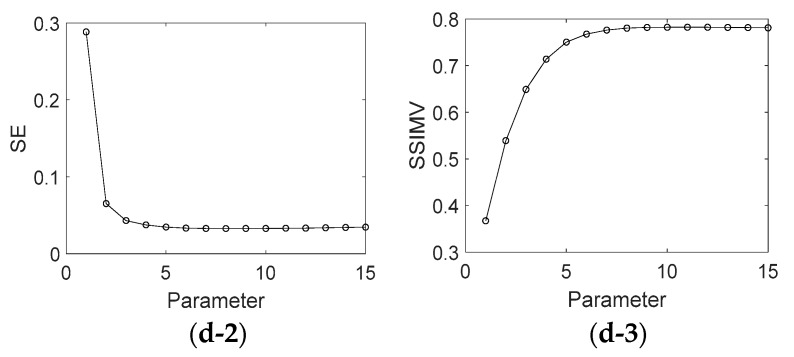
CrossUV, SE and SSIMV for Figure 2 by Fourier transform method with different parameter values. (**a-1**) CrossUV for Figure 2a; (**a-2**) SE for Figure 2a; (**a-3**) SSIMV for Figure 2a; (**b-1**) CrossUV for Figure 2b; (**b-2**) SE for Figure 2b; (**b-3**) SSIMV for Figure 2b. (**c-1**) CrossUV for Figure 2c; (**c-2**) SE for Figure 2c; (**c-3**) SSIMV for Figure 2c; (**d-1**) CrossUV for Figure 2d; (**d-2**) SE for Figure 2d; (**d-3**) SSIMV for Figure 2d.

Like Figure 5, Figure 7 shows the assessment index of CrossUV, SE and SSIMV for simulated fringe patterns (Figure 2) by Fourier transform method under a set of model parameter values. Specifically, Figure 7(a-1)–(a-3) respectively show the CrossUV, SE and SSIMV for Figure 2a; Figure 7(b-1)–(b-3) respectively show the CrossUV, SE and SSIMV for Figure 2b; Figure 7(c-1)–(c-3) respectively show the CrossUV, SE and SSIMV for Figure 2c; Figure 7(d-1)–(d-3) respectively show the CrossUV, SE and SSIMV for Figure 2d.

**Figure 8:**
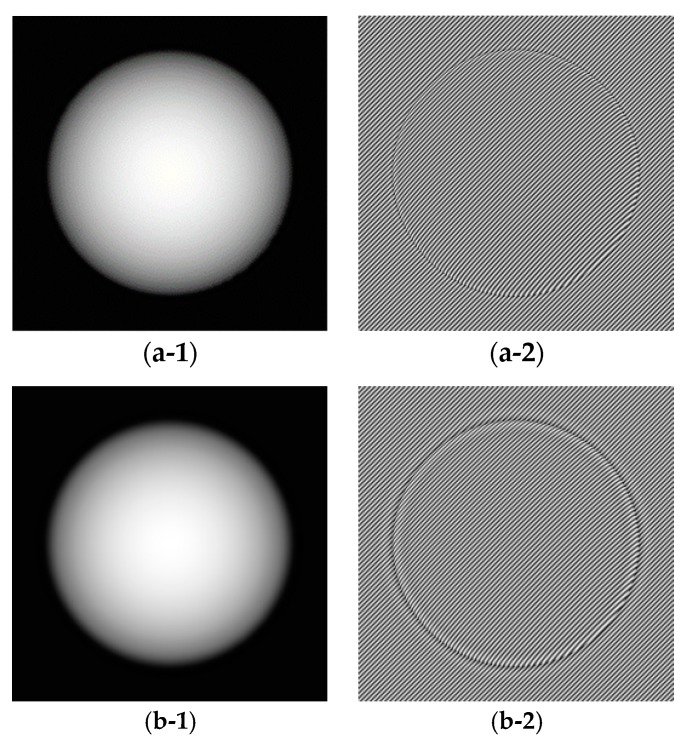

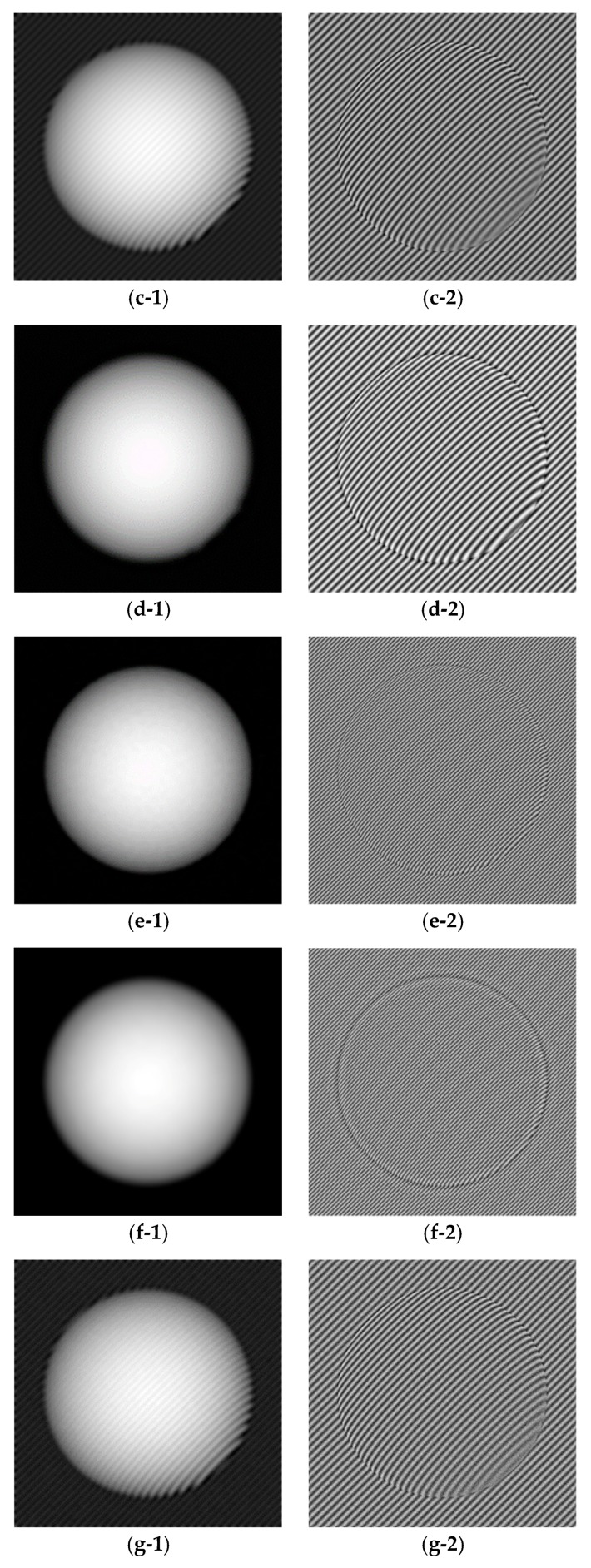

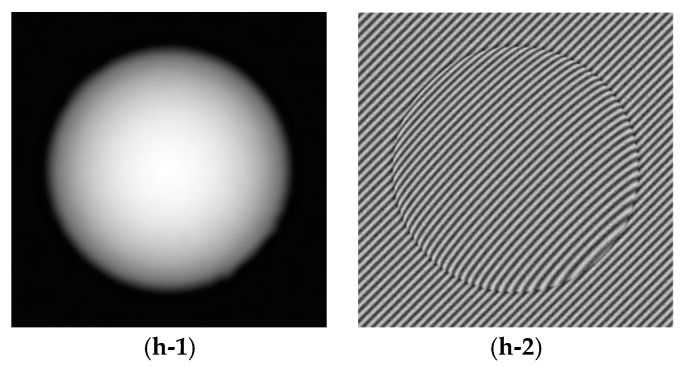
The decomposed background and fringe of Figure 1 by Shearlet transform method with different parameter values. (**a-1**) decomposed background from Figure 1a with decomposition layer 3; (**b-1**) decomposed background from Figure 1a with decomposition layer 4; (**c-1**) decomposed background from Figure 1b with decomposition layer 3; (**d-1**) decomposed background from Figure 1b with decomposition layer 4; (**e-1**) decomposed background from Figure 1c with decomposition layer 3; (**f-1**) decomposed background from Figure 1c with decomposition layer 4; (**g-1**) decomposed background from Figure 1d with decomposition layer 3; (**h-1**) decomposed background from Figure 1d with decomposition layer 4; (**a-2**) decomposed fringe from Figure 1a with decomposition layer 3; (**b-2**) decomposed fringe from Figure 1a with decomposition layer 4; (**c-2**) decomposed fringe from Figure 1b with decomposition layer 3; (**d-2**) decomposed fringe from Figure 1b with decomposition layer 4; (**e-2**) decomposed fringe from Figure 1c with decomposition layer 3; (**f-2**) decomposed fringe from Figure 1c with decomposition layer 4; (**g-2**) decomposed fringe from Figure 1d with decomposition layer 3; (**h-2**) decomposed fringe from Figure 1d with decomposition layer 4.

Figure 8 shows the decomposed background and fringe for Figure 1 by Shearlet transform method with decomposition layer of 3 and 4. In detail, Figure 8(a-1),(b-1) are decomposed background from Figure 1a with decomposition layer 3 and 4, respectively; Figure 8(c-1),(d-1) are decomposed background from Figure 1b with decomposition layer 3 and 4; Figure 8(e-1),(f-1) are decomposed background from Figure 1c with decomposition layer 3 and 4; Figure 8(g-1),(h-1) are decomposed background from Figure 1d with decomposition layer 3 and 4; Figure 8(a-2),(b-2) are decomposed fringes from Figure 1a with decomposition layer 3 and 4, respectively; Figure 8(c-2),(d-2) are decomposed fringes from Figure 1b with decomposition layer 3 and 4; Figure 8(e-2),(f-2) are decomposed fringes from Figure 1c with decomposition layer 3 and 4; Figure 8(g-2),(h-2) are decomposed fringes from Figure 1d with decomposition layer 3 and 4.

**Figure 9:**
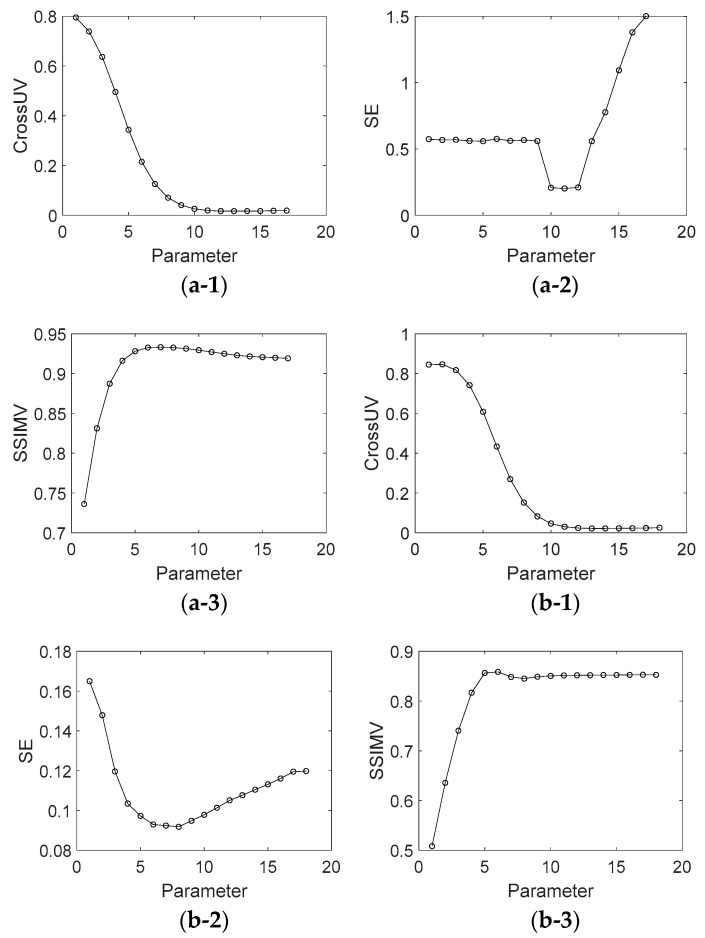
CrossUV, SE and SSIMV for Figure 3 by Fourier transform method with different parameter values. (**a-1**) CrossUV for Figure 3a; (**a-2**) SE for Figure 3a; (**a-3**) SSIMV for Figure 3a; (**b-1**) CrossUV for Figure 3b; (**b-2**) SE for Figure 3b; (**b-3**) SSIMV for Figure 3b.

**Figure 10:**
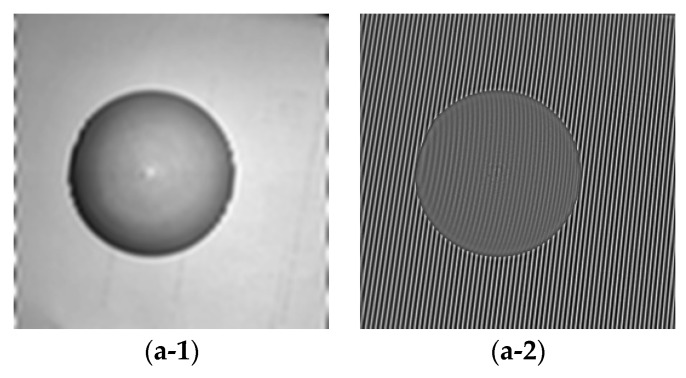

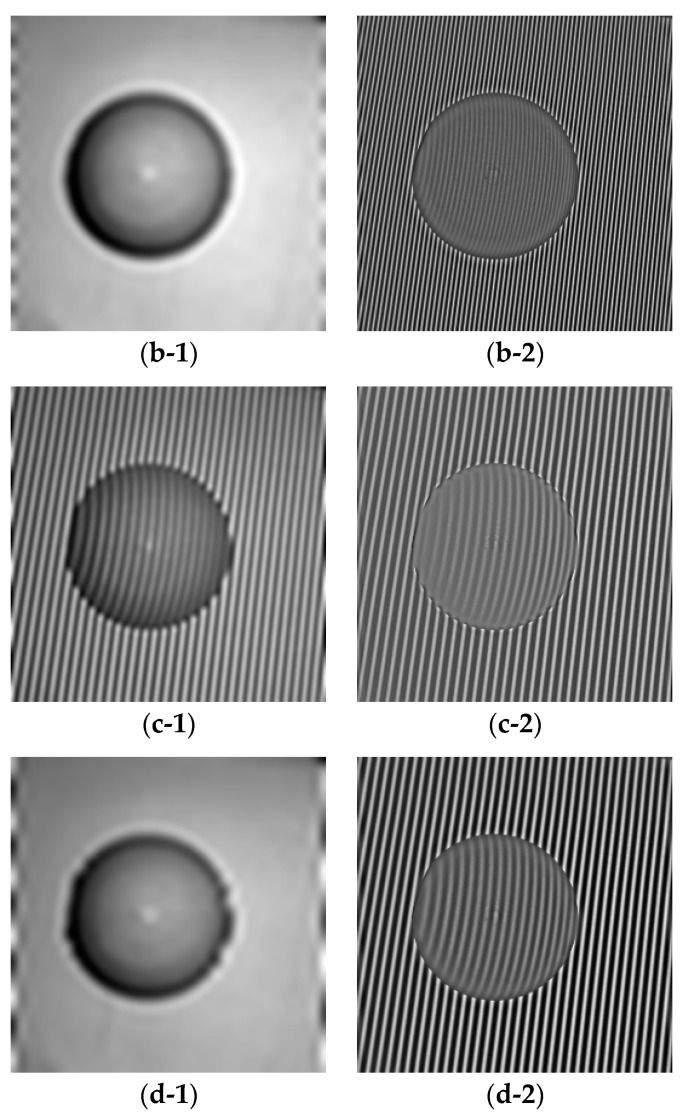
The decomposed background and fringe of Figure 3 by Shearlet transform method with decomposition layer 3 and 4. (**a-1**) decomposed background from Figure 3a with decomposition layer 3; (**b-1**) decomposed background from Figure 3a with decomposition layer 4; (**c-1**) decomposed background from Figure 3b with decomposition layer 3; (**d-1**) decomposed background from Figure 3b with decomposition layer 4; (**a-2**) decomposed fringe from Figure 3a with decomposition layer 3; (**b-2**) decomposed fringe from Figure 3a with decomposition layer 4; (**c-2**) decomposed fringe from Figure 3b with decomposition layer 3; (**d-2**) decomposed fringe from Figure 3b with decomposition layer 4.

**Figure 11:**
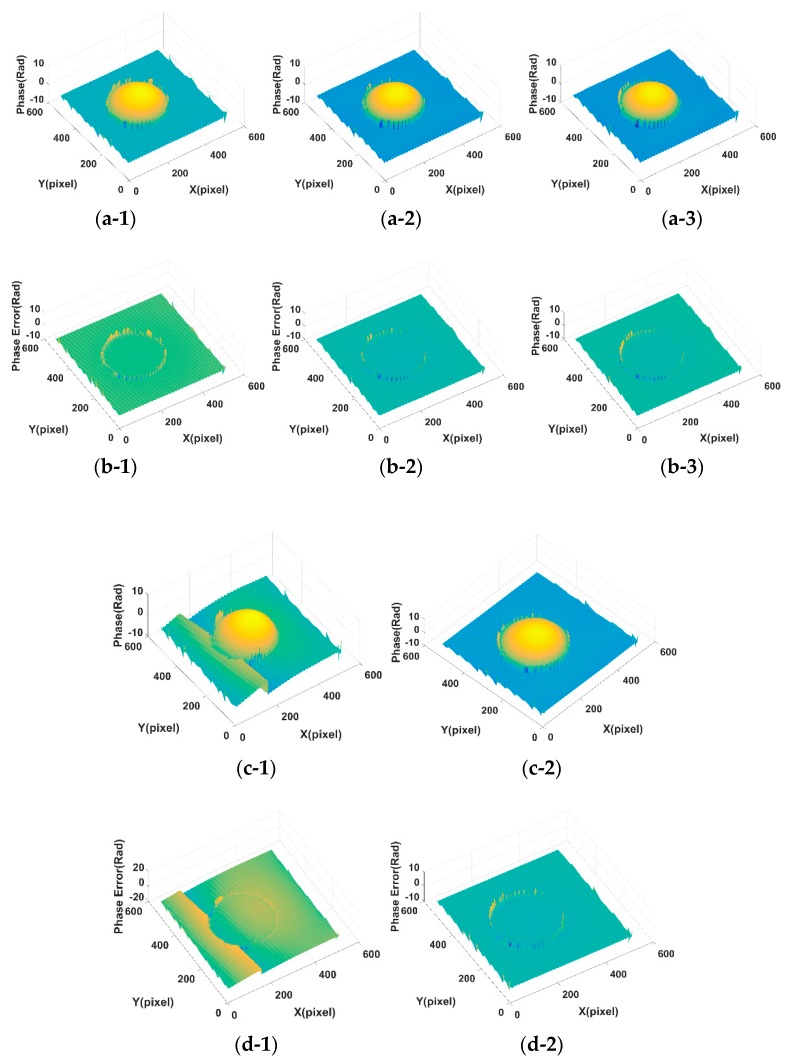
The retrieved phase and phase error for Figure 3b by Fourier transform under optimal CrossUV, SE and SSIMV and Shearlet transform method under different decomposition scales. (**a-1**) Phase under optimal CrossUV for Figure 3b; (**a-2**) Phase under optimal SE for Figure 3b; (**a-3**) Phase under optimal SSIMV for Figure 3b; (**b-1**) Phase error under optimal CrossUV for Figure 3b; (**b-2**) Phase error under optimal SE for Figure 3b; (**b-3**) Phase error under optimal SSIMV for Figure 3b; (**c-1**) Phase under decomposition layer of 3 by Shearlet transform by Figure 3b; (**c-2**) Phase under decomposition layer of 4 by Shearlet transform by Figure 3b; (**d-1**) Phase error under decomposition layer of 3 by Shearlet transform by Figure 3b; (**d-2**) Phase error under decomposition layer of 4 by Shearlet transform by Figure 3b.

**Figure A3:**
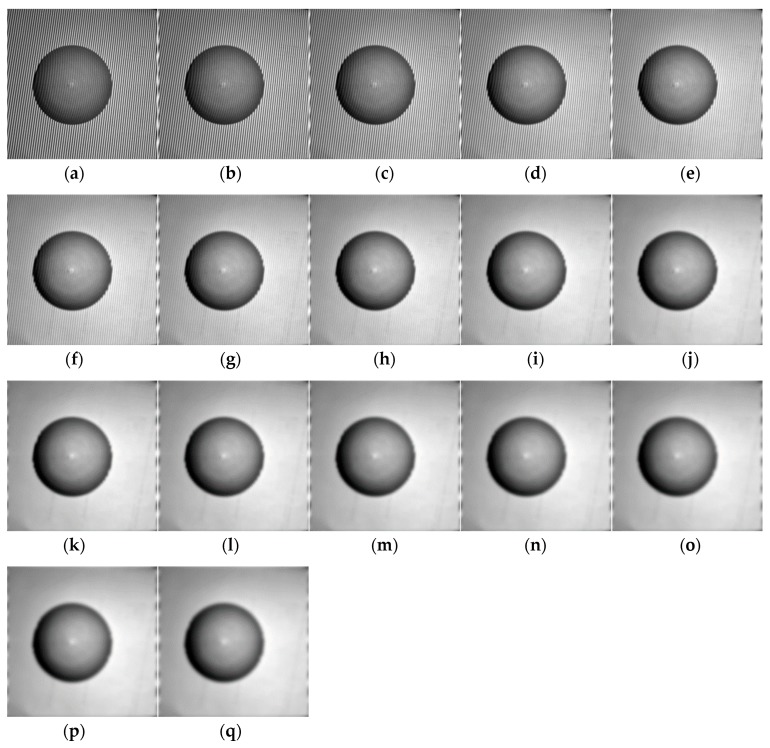
The decomposed background of Figure 3a by Fourier transform method with different parameter values. (**a**–**q**) are the decomposed background by Fourier transform method with 1th to 17th of filtering window sizes of [4:1:20], respectively.

**Figure A4:**
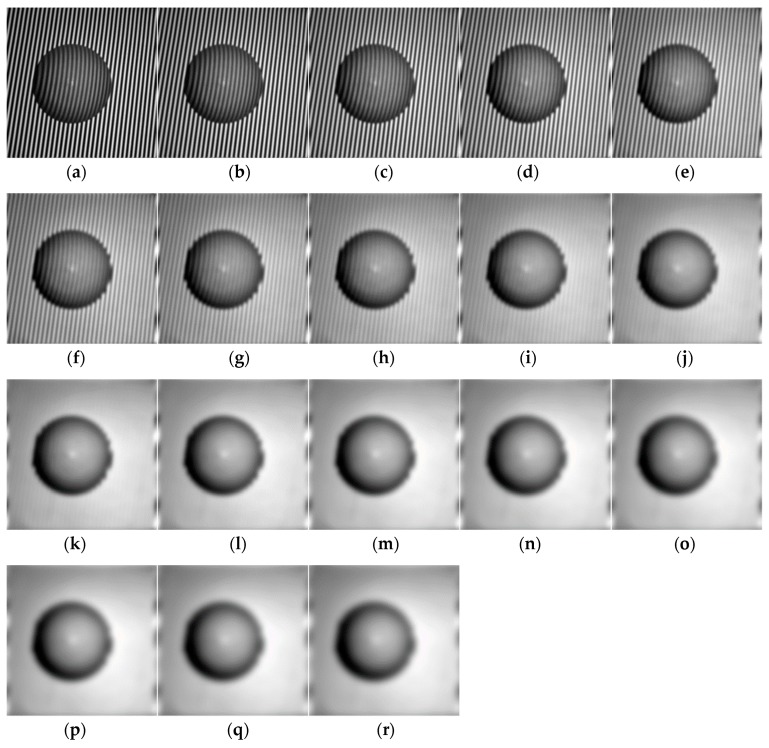
The decomposed background of Figure 3b by Fourier transform method with different parameter values. (**a**–**r**) are the decomposed background by Fourier transform method with 1th to 18th of filtering window sizes of [6:2:40], respectively.

Experiments are carried out as well. Figure A3 and Figure A4 (See Appendix A) show the decomposition background parts of Figure 3 under different parameter values. Similar to Figure 5 and Figure 7, Figure 9 shows the assessment index of CrossUV, SE and SSIMV for Figure 3 by the Fourier transform method. Figure 10 shows the decomposed background and fringe for Figure 3 by the Shearlet transform method with decomposition layers of 3 and 4. Figure 11 shows the retrieved phase and phase error for Figure 3b by the Fourier transform under optimal CrossUV, SE and SSIMV and Shearlet transform method under different decomposition scales.

Table 1 shows optimal CrossUV, SE, and SSIMV computed from simulated and experimental fringe patterns by Fourier transform method. The positions of optimal CrossUV, SE, and SSIMV are shown in the plots of Figure 5, Figure 7 and Figure 9 in the case of the minimal CrossUV, SE and maximal SSIMV. Table 2 shows the CrossUV, SE and SSIMV computed from the decomposed simulated and experimental fringe patterns by Shearlet transform method with decomposition layer 3 and 4. Specially, the CrossUV, and SSIMV in Table 2 are from the decomposed fringe parts in Figure 8 and Figure 10, and the SE in Table 2 from the retrieved phase in Figure 11.

As shown in Figure A1, Figure A2, Figure A3 and Figure A4, the decomposition result of projection fringe patterns varies according to the value of model parameter for Fourier transform. Figure 5, Figure 7 and Figure 9 show that the cross-correlation of fringes and background firstly decreases and then increases with the increased parameter values of Fourier transform. Moreover, SE of unwrapped phase also firstly decreases and then increases. In contrast, the SSIM of fringes parts (SSIMV) firstly increases and then decreases. It is known that the minimal value of SE and cross-correlation is optimal while the maximum value of SSIM is optimal. These results from simulated and experimental data suggest that the decomposition results are related to the values of parameter, and they become better and then become worse with the continuous increasing parameter values. Therefore, it is important to choose the appropriate value of model parameter to achieve desirable results.

Further, it can be drawn that the parameter with minimal cross-correlation is generally consist that with the minimal SE and SSIMV. The optimal decomposed results also show this accordance. For instance, the optimal SE and CrossUV in Table 1 for Figure 1b are both under the 8th parameter. Also, the optimal SSIMV exists at the 8th parameter value. With these, we can conclude that the quality of decomposition results can be assessed by cross-correlation of decomposed fringe and background, i.e., smaller cross-correlation metric corresponds to better decomposition results and phase retrieval results.

For the Shearlet transform, as shown in Figure 8, Figure 10 and Figure 11 as well as Table 2, the decomposition results vary with different decomposition layers. Taking the results for Figure 3b for example, the decomposed background with a decomposition layer of 3 still contains a lot of fringes as shown in Figure 10(c-1). The results lead to a larger cross-correlation metric of 7.16 × 10^−1^ as illustrated in Table 2, compared to 4.10 × 10^−3^ which is obtained from the decomposition with decomposition layer of 4. Overall, decomposition results with decomposition scale 3, in terms of CrossUV, SE and SSIM, is better than that from decomposition results with decomposition scale 4 for the fringe patterns with a large frequency, which are fringe patterns with a small frequency. On the contrary, the decomposition result with decomposition scale 4, in terms of CrossUV, SE and SSIM, is better than that with decomposition scale 3 for a small frequency, which are fringe patterns with small frequency. It is also seen that the minimal CrossUV, SE, and maximal SSIM are at the same decomposition scale, which demonstrates that the index of cross-correlation is able to assess the decomposition results.

It is also noted that while 2D image quality assessment has been an active research topic, 3D image quality assessment is more difficult and lacks new quality metrics. On one hand, we only use the SE to assess unwrapped phases (3D data). On the other hand, phase unwrapping is a difficult problem leading because the unwrapped phase is sensitive to the decomposed fringe. As shown in Table 1, the optimal parameter position with respect to cross-correlation, to some extent, deviates from that with SE of unwrapped phase and SSIM of decomposed fringe. This deviation might be related to the accuracy of unwrapped phases, or the accuracy of retrieved wrapped phase. The future work is to reduce the deviation by considering these two issues. However, cross-correlation of decomposed background and fringe generally indicates quality of the decomposition results and phase result quality and can be used as an assessment index for decomposition.

## 4. Conclusions

In this work, we have conducted a performance assessment of the decomposition results of fringe patterns under different parameter values and proposed a cross-correlation metric index to assess the decomposition results. The results from both the Fourier transform and Shearlet transform methods demonstrate that an optimal (minimal) cross-correlation index exists under a set of parameter values. The optimal decomposition for fringe pattern in terms of cross-correlation is verified by the decomposed fringe with SSIM index, and is also verified by the unwrapped phase with SE index. Our proposed cross-correlation metric for the assessment of the decomposition results without the need of ground truth data is simple, and yet feasible in the application of automatic parameter selection in phase retrieval methods of Fourier transform and Shearlet transform, and may be extended to WFT and EMD, etc. Future work will focus on the improvement of the proposed method in the application of Fourier transform method with respect to the assessment accuracy and speed. This work should prove beneficial for the automatic 3D fringe projection measurement with less human intervention, and could be extended to other fields such as phase retrieval in digital holography.

## Figures and Tables

**Table 1:** Optimal CrossUV, SE, and SSIMV computed from simulated and experimental fringe patterns by the Fourier transform method.

Figures	CrossUV	SE	SSIMV
Figure 1a	6.70 × 10^−3^ (10th)	6.72 × 10^−3^ (5th)	9.68 × 10^−1^ (8th)
Figure 1b	1.99 × 10^−2^ (8th)	2.09 × 10^−2^ (8th)	9.07 × 10^−1^ (8th)
Figure 1c	6.93 × 10^−3^ (10th)	1.55 × 10^−2^ (7th)	8.56 × 10^−1^ (8th)
Figure 1d	1.92 × 10^−2^ (9th)	2.23 × 10^−2^ (9th)	7.35 × 10^−1^ (8th)
Figure 2a	7.96 × 10^−3^ (14th)	2.58 × 10^−2^ (13th)	9.99 × 10^−1^ (12th)
Figure 2b	3.06 × 10^−2^ (10th)	2.75 × 10^−2^ (8th)	9.95 × 10^−1^ (8th)
Figure 2c	1.03 × 10^−2^ (15th)	3.63 × 10^−2^ (10th)	7.97 × 10^−1^ (11th)
Figure 2d	3.19 × 10^−2^ (10th)	3.27 × 10^−2^ (8th)	7.82 × 10^−1^ (11th)
Figure 3a	1.62 × 10^−2^ (13th)	2.02 × 10^−1^ (11th)	9.33 × 10^−1^ (7th)
Figure 3b	2.17 × 10^−2^ (14th)	9.18 × 10^−2^ (8th)	8.59 × 10^−1^ (6th)

**Table 2:** CrossUV, SE and SSIMV computed from the decomposed simulated and experimental fringe patterns by Shearlet transform method with different parameter values. The optimal values are marked in bold.

Figures	Shearlet (3 Scales)			Shearlet (4 Scales)		
	CrossUV	SE	SSIMV	CrossUV	SE	SSIMV
Figure 1a	**2.62 × 10^−4^**	**6.51 × 10^−3^**	**9.73 × 10^−1^**	1.04 × 10^−3^	9.19 × 10^−3^	9.53 × 10^−1^
Figure 1b	5.30 × 10^−2^	5.38 × 10^−2^	8.26 × 10^−1^	**1.01 × 10^−3^**	**1.86 × 10^−2^**	**9.19 × 10^−1^**
Figure 1c	**3.01 × 10^−4^**	**1.48 × 10^−2^**	**8.16 × 10^−1^**	9.18 × 10^−4^	1.63 × 10^−2^	7.96 × 10^−1^
Figure 1d	5.07 × 10^−2^	5.31 × 10^−2^	7.01 × 10^−1^	**1.08 × 10^−3^**	**1.98 × 10^−2^**	**7.41 × 10^−1^**
Figure 3a	**2.76 × 10^−3^**	**1.78 × 10^−1^**	**9.29 × 10^−1^**	4.68 × 10^−3^	8.17 × 10^−1^	9.15 × 10^−1^
Figure 3b	7.16 × 10^−1^	5.64 × 10^−1^	7.01 × 10^−1^	**4.10 × 10^−3^**	**9.18 × 10^−2^**	**8.66 × 10^−1^**

## Appendix A

Figure A1 shows the decomposed results for Figure 1a by the Fourier transform method with different parameter values. Subfigures (a–s) are the backgrounds decomposed by the Fourier transform method with 1st to 19th filtering window sizes of [2:1:20], respectively. Figure A2 shows the decomposition results for Figure 1b by the Fourier transform method with different parameters. Subfigures (a–n) are the backgrounds decomposed by the Fourier transform method with 1st to 14th filtering window sizes of [4:2:30], respectively. Figure A3 shows the decomposition results for Figure 3a by the Fourier transform method with different parameter values. Subfigures (a–q) are the decomposed backgrounds by the Fourier transform method with 1st to 17th filtering window sizes of [4:1:20], respectively. Figure A4 shows the decomposition results for Figure 3b by the Fourier transform method with different parameter values. Subfigures (a–r) are the decomposed backgrounds by the Fourier transform method with 1st to 18th of filtering window sizes of [6:2:40], respectively.
